# Supplementary material for: HIVprotI: an integrated web based platform for prediction and design of HIV proteins inhibitors
Source: J Cheminform. 2018 Mar 9;10:12. doi: 10.1186/s13321-018-0266-y (PMC5845081; doi:10.1186/s13321-018-0266-y)
Supplement: Supplementary file 2 — Additional file 2. Source code of HIVProtI web server. [file 13321_2018_266_MOESM2_ESM.zip › HIVprotI_Source-code/hivprotI/weka-3-6-6/doc/weka/classifiers/functions/supportVector/KernelEvaluation.html]

KernelEvaluation


---


|  |  |  |
| --- | --- | --- |
| **Overview** \| **Package** \| **Class** \| **Tree** \| **Deprecated** \| **Index** \| **Help** \| **Weka's home** | |  |
| **PREV CLASS**   **NEXT CLASS** | **FRAMES**    **NO FRAMES**     **All Classes** |  |
| SUMMARY: NESTED \| FIELD \| CONSTR \| METHOD | DETAIL: FIELD \| CONSTR \| METHOD |  |


---


## weka.classifiers.functions.supportVector Class KernelEvaluation

```
java.lang.Object
  weka.classifiers.functions.supportVector.KernelEvaluation
```

**All Implemented Interfaces:**: RevisionHandler

---

``` public class KernelEvaluation extends java.lang.Object implements RevisionHandler ```

Class for evaluating Kernels.

**Version:**
:   $Revision: 1.3 $

**Author:**
:   fracpete (fracpete at waikato dot ac dot nz)

---

**Constructor Summary**

`KernelEvaluation()`
  
          default constructor


| **Method Summary** | |
| --- | --- |
| `boolean` | `equals(java.lang.Object obj)`             Tests whether the current evaluation object is equal to another evaluation object |
| `java.lang.String` | `evaluate(Kernel kernel, Instances data)`             Evaluates the Kernel with the given commandline options and returns the evaluation string. |
| `static java.lang.String` | `evaluate(Kernel Kernel, java.lang.String[] options)`             Evaluates the Kernel with the given commandline options and returns the evaluation string. |
| `static java.lang.String` | `evaluate(java.lang.String kernelString, java.lang.String[] options)`             Evaluates a kernel with the options given in an array of strings. |
| `java.lang.String` | `getRevision()`             Returns the revision string. |
| `java.lang.String[]` | `getUserOptions()`             returns the options the user supplied for the kernel |
| `static void` | `main(java.lang.String[] args)`             A test method for this class. |
| `void` | `setUserOptions(java.lang.String[] options)`             sets the option the user supplied for the kernel |
| `java.lang.String` | `toString()`             returns the current result |
| `java.lang.String` | `toSummaryString()`             returns a summary string of the evaluation with a no title |
| `java.lang.String` | `toSummaryString(java.lang.String title)`             returns a summary string of the evaluation with a default title |

**Methods inherited from class java.lang.Object**

`getClass, hashCode, notify, notifyAll, wait, wait, wait`

**Constructor Detail**

### KernelEvaluation

```
public KernelEvaluation()
```

:   default constructor


**Method Detail**

### setUserOptions

```
public void setUserOptions(java.lang.String[] options)
```

:   sets the option the user supplied for the kernel

    :   **Parameters:**: `options` - options that were supplied for the kernel

---


### getUserOptions

```
public java.lang.String[] getUserOptions()
```

:   returns the options the user supplied for the kernel

    :   **Returns:**: the user supplied options for the kernel

---


### evaluate

```
public static java.lang.String evaluate(Kernel Kernel,
                                        java.lang.String[] options)
                                 throws java.lang.Exception
```

:   Evaluates the Kernel with the given commandline options and returns
    the evaluation string.

    :   **Parameters:**: `Kernel` - the Kernel to evaluate: `options` - the commandline options **Returns:**: the generated output string **Throws:**: `java.lang.Exception` - if evaluation fails

---


### evaluate

```
public static java.lang.String evaluate(java.lang.String kernelString,
                                        java.lang.String[] options)
                                 throws java.lang.Exception
```

:   Evaluates a kernel with the options given in an array of strings.

    :   **Parameters:**: `kernelString` - class of kernel as a string: `options` - the array of string containing the options **Returns:**: a string describing the results **Throws:**: `java.lang.Exception` - if model could not be evaluated successfully

---


### evaluate

```
public java.lang.String evaluate(Kernel kernel,
                                 Instances data)
                          throws java.lang.Exception
```

:   Evaluates the Kernel with the given commandline options and returns
    the evaluation string.

    :   **Parameters:**: `kernel` - the Kernel to evaluate: `data` - the data to run the Kernel with **Returns:**: the generated output string **Throws:**: `java.lang.Exception` - if evaluation fails

---


### equals

```
public boolean equals(java.lang.Object obj)
```

:   Tests whether the current evaluation object is equal to another
    evaluation object

    :   **Overrides:**: `equals` in class `java.lang.Object`
    :   **Parameters:**: `obj` - the object to compare against **Returns:**: true if the two objects are equal

---


### toSummaryString

```
public java.lang.String toSummaryString()
```

:   returns a summary string of the evaluation with a no title

    :   **Returns:**: the summary string

---


### toSummaryString

```
public java.lang.String toSummaryString(java.lang.String title)
```

:   returns a summary string of the evaluation with a default title

    :   **Parameters:**: `title` - the title to print before the result **Returns:**: the summary string

---


### toString

```
public java.lang.String toString()
```

:   returns the current result

    :   **Overrides:**: `toString` in class `java.lang.Object`
    :   **Returns:**: the currently stored result **See Also:**: `toSummaryString()`

---


### getRevision

```
public java.lang.String getRevision()
```

:   Returns the revision string.

    :   **Specified by:**: `getRevision` in interface `RevisionHandler`
    :   **Returns:**: the revision

---


### main

```
public static void main(java.lang.String[] args)
```

:   A test method for this class. Just extracts the first command line
    argument as a kernel class name and calls evaluate.

    :   **Parameters:**: `args` - an array of command line arguments, the first of which must be the class name of a kernel.


---


|  |  |  |
| --- | --- | --- |
| **Overview** \| **Package** \| **Class** \| **Tree** \| **Deprecated** \| **Index** \| **Help** \| **Weka's home** | |  |
| **PREV CLASS**   **NEXT CLASS** | **FRAMES**    **NO FRAMES**     **All Classes** |  |
| SUMMARY: NESTED \| FIELD \| CONSTR \| METHOD | DETAIL: FIELD \| CONSTR \| METHOD |  |


---
